# Amyloid β production is regulated by β2-adrenergic signaling-mediated post-translational modifications of the ryanodine receptor

**DOI:** 10.1074/jbc.M116.743070

**Published:** 2017-05-05

**Authors:** Renaud Bussiere, Alain Lacampagne, Steven Reiken, Xiaoping Liu, Valerie Scheuerman, Ran Zalk, Cécile Martin, Frederic Checler, Andrew R. Marks, Mounia Chami

**Affiliations:** From the ‡ Université Côte d'Azur, CNRS, IPMC, France, “Labex Distalz,” 660 route des Lucioles, 06560 Sophia-Antipolis, Valbonne, France,; § INSERM U1046, CNRS UMR9214, CNRS LIA1185, Université de Montpellier, CHRU Montpellier, 34295 Montpellier, France, and; ¶ Department of Physiology and Cellular Biophysics, Clyde and Helen Wu Center for Molecular Cardiology, Columbia University College of Physicians and Surgeons, New York, New York 10032

**Keywords:** Alzheimer disease, amyloid precursor protein (APP), amyloid-β (AB), calcium intracellular release, ryanodine receptor, β 2 adrenergic signaling, calstabin2

## Abstract

Alteration of ryanodine receptor (RyR)-mediated calcium (Ca^2+^) signaling has been reported in Alzheimer disease (AD) models. However, the molecular mechanisms underlying altered RyR-mediated intracellular Ca^2+^ release in AD remain to be fully elucidated. We report here that RyR2 undergoes post-translational modifications (phosphorylation, oxidation, and nitrosylation) in SH-SY5Y neuroblastoma cells expressing the β-amyloid precursor protein (βAPP) harboring the familial double Swedish mutations (APPswe). RyR2 macromolecular complex remodeling, characterized by depletion of the regulatory protein calstabin2, resulted in increased cytosolic Ca^2+^ levels and mitochondrial oxidative stress. We also report a functional interplay between amyloid β (Aβ), β-adrenergic signaling, and altered Ca^2+^ signaling via leaky RyR2 channels. Thus, post-translational modifications of RyR occur downstream of Aβ through a β2-adrenergic signaling cascade that activates PKA. RyR2 remodeling in turn enhances βAPP processing. Importantly, pharmacological stabilization of the binding of calstabin2 to RyR2 channels, which prevents Ca^2+^ leakage, or blocking the β2-adrenergic signaling cascade reduced βAPP processing and the production of Aβ in APPswe-expressing SH-SY5Y cells. We conclude that targeting RyR-mediated Ca^2+^ leakage may be a therapeutic approach to treat AD.

## Introduction

Alzheimer disease (AD)^5^ is one of the leading neurodegenerative pathologies in the western countries. The two main neuropathological lesions of AD are amyloid plaques, composed mainly of amyloid β (Aβ) peptides, and neurofibrillary tangles, composed of hyperphosphorylated tau (1, 2). The Aβ peptides, which form the core of the amyloid plaques, are produced by the sequential proteolytic cleavages of the β amyloid precursor protein (βAPP). βAPP can be processed by two alternative post-translational pathways: (i) an amyloidogenic route in which βAPP is cleaved by β-secretase (BACE-1) to generate a soluble sAPPβ fragment and a C-terminal fragment of 99 amino acids (C99), which is further cleaved by a presenilin (PS1 and PS2)-dependent γ-secretase complex to generate Aβ and AICD (APP intracellular domain) and (ii) a non-amyloidogenic pathway in which βAPP is sequentially hydrolyzed by α-secretase to produce a soluble sAPPα and a C-terminal fragment of 83 amino acids (C83). C83 is further cleaved by γ-secretase to generate a p3 peptide and AICD (3, 4). The amyloid cascade hypothesis is mainly supported by genetic studies indicating that autosomal dominant cases of AD (familial AD) are linked to mutations in βAPP (5) and on PS1 and PS2 (6, 7), leading to modifications of Aβ production.

Data are now converging to suggest an important contribution of endoplasmic reticulum (ER) Ca^2+^ homeostasis deregulation in AD pathological process (8, 9). This combines pathological ER Ca^2+^ release via the inositol 1,4,5-trisphosphate (IP_3_R) (10–12), and the ryanodine (RyR) receptors (9, 13–16). Importantly, alteration of RyR-mediated Ca^2+^ release likely contributes to ER Ca^2+^ deregulation in AD (9).

RyR dysfunction has been reported in AD models; however, the molecular mechanisms underlying RyR-mediated ER Ca^2+^ leak in AD are still not fully understood. RyR macromolecular complexes include four RyR protomers (565 kDa each) and several regulatory proteins including the RyR channel-stabilizing subunit calstabin. Recent studies have revealed that RyR-mediated ER Ca^2+^ depletion is linked to post-translational modifications (hypernitrosylation, hyperphosphorylation, oxidation) of the RyR macromolecular complexes resulting in calstabin depletion and “leaky RyR channels” (17–19).

β2-Adrenergic receptors (β2-ARs) have been implicated in the development of AD both in humans and in AD animal models (20–23). However, the association between β-adrenergic signaling and RyR-mediated Ca^2+^ deregulation in AD has not been reported.

## Results

### Remodeling of RyR2 macromolecular complexes in SH-SY5Y neuroblastoma cells stably expressing APPswe

We examined post-translational modifications of RyR2 channels consistent with the biochemical signature of leaky RyR2 channels (19, 24–28) in an *in vitro* AD study model. We used human SH-SY5Y neuroblastoma cells stably expressing pcDNA3.1 (control) or human βAPP harboring the double Swedish mutations (APPswe: APPKM670/671NL) constructs. We previously reported that SH-SY5Y cells expressing APPswe yield increased levels of APP C-terminal fragments (CTFs) fragments (C99 and C83) and of Aβ peptides (13).

**Figure 1.**
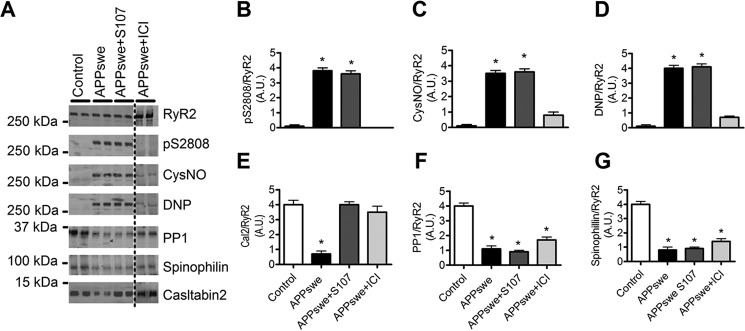
**Remodeling of RyR2 macromolecular complex in SH-SY5Y neuroblastoma cell line stably expressing APPswe.** *A*, representative SDS-PAGE analyses and quantification of RyR2 immunoprecipitated from the human SH-SY5Y neuroblastoma cell line stably expressing APPswe or mock vector used as control (*Control*). RyR2 was immunoprecipitated and immunoblotted for RyR2-pS2808 (RyR2 PKA phosphorylation site), *S*-nitrosylation (*CysNO*) and oxidation (*DNP*) as well as components of the RyR2 channel complex including: protein phosphatase 1 (*PP1*) and its anchoring protein spinophilin and calstabin2. Cells were treated with either S107 (10 μm, for 12 h) or ICI118–551 (*ICI*) (1 μm, for 12 h). APPswe+ICI treatment was conducted in the same set of experiments as the control, APPswe, and APPswe+S107 but was run on a separate membrane. *B–G*, graphs represent the mean ± S.E. obtained from three independent experiments. *, *p* < 0.05 calculated *versus* control using one-way ANOVA and Bonferroni post-test. *A.U.*, arbitrary units.

RyR2 was immunoprecipitated and immunoblotted for protein kinase A (PKA) phosphorylation (at residue Ser-2808), oxidation (2,4-dinitrophenylhydrazone (DNP)), nitrosylation (anti-Cys NO), and levels of the channel stabilizing subunit calstabin2 (FKBP12.6) in the RyR2 macromolecular complex. Neuronal RyR2 from control SH-SY5Y cells had no biochemical remodeling of the RyR2 macromolecular complex, whereas APPswe-expressing cells exhibited RyR2 PKA phosphorylation, oxidation, nitrosylation, and calstabin2 depletion (Fig. 1, *A–E*). RyR2 macromolecular complexes from APPswe-expressing cells also exhibited reduced levels of serine/threonine protein phosphatase 1 (PP1) and spinophilin (Fig. 1, *A*, *F*, and *G*) (24, 25). We then tested whether the Rycal S107 can prevent depletion of calstabin2 from RyR2 (19, 26–28). S107 had no effect on PKA phosphorylation or oxidation/nitrosylation of RyR2 (Fig. 1, *A–D*) but significantly prevented dissociation of calstabin2 from RyR2 channels (Fig. 1, *A* and *E*). S107 did not restore PP1 and spinophilin levels in RyR2 complexes (Fig. 1, *A*, F, and *G*). PKA phosphorylation of RyR2 occurs downstream of β2-AR activation (24, 25). In addition, we already reported that PKA, H_2_O_2_, and Noc-12 (NO-donor) individually caused a ∼2-fold and, in combination, caused an ∼4-fold decrease in the binding affinity of calstabin2 to neuronal RyR2 (19). Because the β2-AR has been reported to trigger AD-related biochemical and anatomical alterations (20–23), we examined the effects of β2-AR blockade on RyR2 post-translational modifications and remodeling. The selective β2-AR antagonist ICI118–551 (ICI) prevented RyR2 PKA phosphorylation, oxidation, nitrosylation, and depletion of calstabin2 (Fig. 1, *A–E*) without affecting PP1 and spinophilin levels on RyR2 complex (Fig. 1, *A*, F, and *G*).

**Figure 2.**
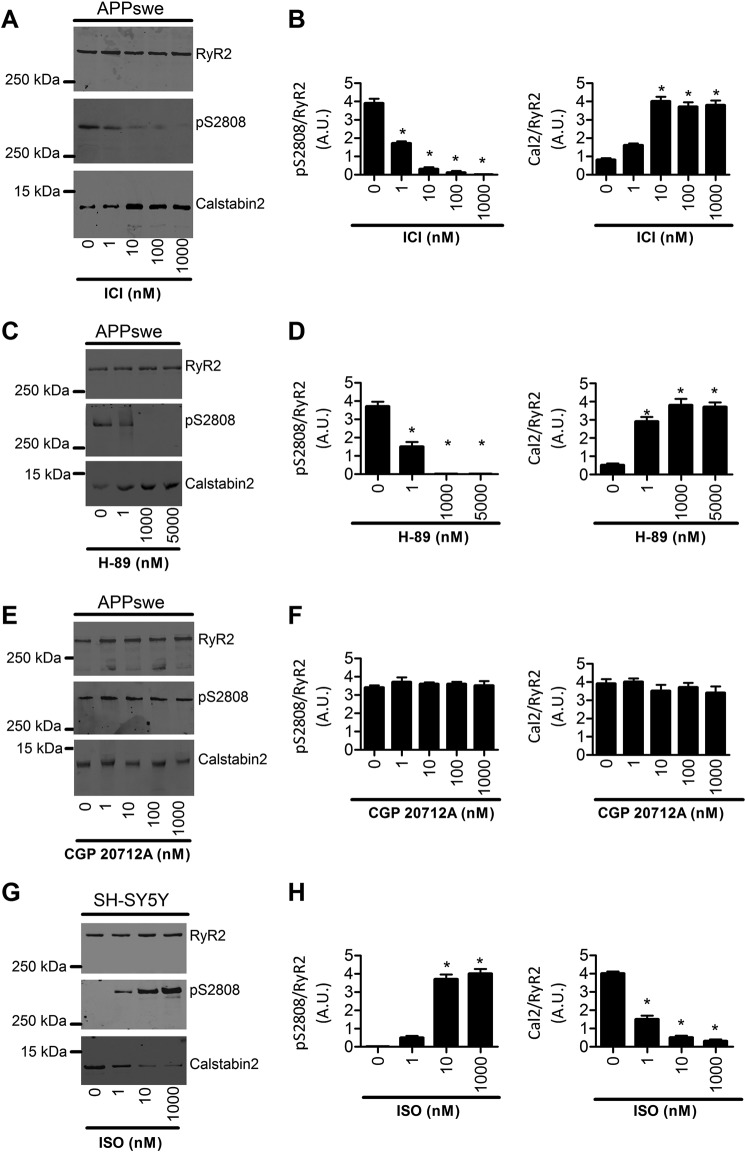
**Remodeling of the RyR2 macromolecular complex in the SH-SY5Y neuroblastoma cell line stably expressing APPswe and in SH-SY5Y control cells.** Representative SDS-PAGE analyses and quantification of RyR2 immunoprecipitated from the human SH-SY5Y neuroblastoma cell line stably expressing APPswe treated with different doses of ICI118–551 (*ICI*) (1 nm, 10 nm, 100 nm, and 1000 nm, for 12 h) (*A*), H-89 (1 nm, 1000 nm, and 5000 nm, for 12 h) (*C*), and CGP 20217A (1 nm, 10 nm, 100 nm, and 1000 nm, for 12 h) (*E*), and from SH-SY5Y control cells treated with different doses of Isoproterenol (1 nm, 10 nm, and 1000 nm, for 1 h) (*G*). RyR2 was immunoprecipitated and immunoblotted for RyR2-pS2808 (RyR2 PKA phosphorylation site) and for calstabin2. Graphs *B*, *D*, *F*, and *H* represent the mean ± S.E. obtained from three independent experiments. *, *p* < 0.05 calculated *versus* control using one-way ANOVA and Bonferroni post-test. *A.U.*, arbitrary units.

We further performed several tests to ascertain that RyR2 post-translational remodeling occurs through β2-AR in our study model. Indeed, SH-SY5Y cells express both β1- and β2-AR but not β3-AR (29). APPswe cells were treated using increasing concentrations of the β2-AR antagonist ICI (Fig. 2, *A* and *B*), the PKA inhibitor (H-89) (Fig. 2, *C* and *D*), or the β1-AR antagonist CGP 20712A (Fig. 2, *E* and *F*). We show that complete blockade of RyR2 phosphorylation and calstabin2 dissociation from RyR2 was obtained with ICI at 10 nm, 0.1 μm, and 1 μm (Fig. 2, *A* and *B*). Accordingly, we also observed a dose-dependent blockade of RyR2 post-translational remodeling in APPswe cells with H-89 with a maximal effect at 1 μm and 5 μm (Fig. 2, *C* and *D*). Importantly, treatment of APPswe cells with the β1-AR antagonist CGP 20712A up to 1 μm did not modify either RyR2 phosphorylation or calstabin2 binding to RyR2 (Fig. 2, *E* and *F*). Although H-89 does not behave as a fully specific inhibitor of PKA (30), it supports our view that RyR2 post-translational remodeling in APPswe-expressing cells occurs specifically and in a dose-dependent manner through β2-AR activation (Fig. 2, *A–F*).

### Aβ causes the biochemical signature of leaky RyR2 channels

**Figure 3.**
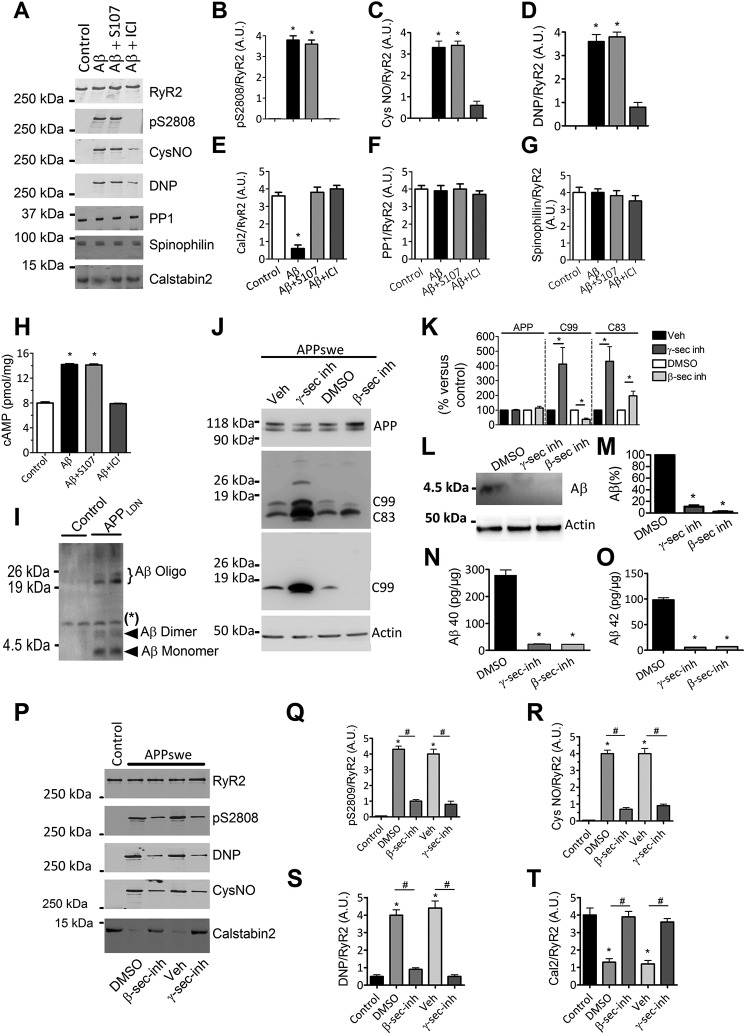
**Aβ caused the biochemical signature of leaky RyR2 channels.** *A*, representative SDS-PAGE analyses and quantification of RyR2 immunoprecipitated from the wild-type human SH-SY5Y neuroblastoma cell line treated with oligomeric Aβ (1–5 nm, 30–60 min) alone or in combination with either S107 (10 μm, for 12 h) or ICI118–551 (*ICI*) (1 μm, for 12 h). RyR2 was immunoprecipitated and immunoblotted for RyR2-pS2808 (RyR2 PKA phosphorylation site), *S*-nitrosylation (*CysNO*), and oxidation (*DNP*) as well as components of the RyR2 channel complex as in Fig. 1. *B–G*, graphs represent the mean ± S.E. obtained from three independent experiments. *, *p* < 0.05 calculated *versus* SH-SY5Y untreated cells used as control (*Control*) using one-way ANOVA and Bonferroni post-test. *H*, Aβ treatment (1–5 nm) for 12 h caused a rise in intracellular [cAMP] (pmol/mg of total proteins) in the SH-SY5Y neuroblastoma cell line that was not reduced by S107 (10 μm, for 12 h) and reduced by ICI (1 μm, for 12 h) (*n* = 5 for each condition). Data are the mean ± S.E. *, *p* < 0.05 calculated *versus Control* using one-way ANOVA and Bonferroni post-test. *I*, a representative Tris-Tricine gel showing oligomeric Aβ preparations obtained from conditioned media of CHO cells stably transfected with hAPP695 cDNA harboring London mutation (APP_LDN_: APPV642I). Conditioned media of CHO cells stably transfected with pcDNA4 empty vector were used as control. *, nonspecific band. *J*, representative SDS-PAGE and Tris-Tricine gels showing the modulation of βAPP metabolism in SH-SY5Y cells expressing APPswe treated for 12 h with β-secretase inhibitor (*inh*; LY288672, 30 μm, for 12 h; Ref. 32) or γ-secretase inhibitor (ELND006, 5 μm, for 12 h; Refs. 33 and 34) or with DMSO (control for β-secretase inhibitor) or vehicle (control for γ-secretase inhibitor) and revealed on total extracts. βAPP was detected using the APP N-terminal antibody. β-CTF (C99) was detected using the 6E10 antibody. α- and β-CTFs (C83 and C99, respectively) were detected using the APP C-terminal antibody. Actin was used as loading control. *K*, graph represents the mean ± S.E. of APP, C99, and C83 obtained from 5–8 independent experiments as shown in *J*. *, *p* < 0.05 calculated *versus* controls (DMSO or vehicle) using one-way ANOVA and Tukey's multiple comparisons test. *L*, representative Tris-Tricine gel showing total intracellular Aβ in the SH-SY5Y neuroblastoma cell line stably expressing APPswe, control (DMSO), or treated for 12 h with γ- or β-secretase inhibitors as in *J*. Actin was used as loading control. *M*, the graph represents the mean ± S.E. obtained from 3 independent experiments. *, *p* < 0.01 calculated *versus* DMSO treated SH-SY5Y APPswe cells using one-way ANOVA and Tukey's multiple comparisons test. *N* and *O*, ELISA of Aβ40 (*N*) and of Aβ42 (*O*) done on cell culture media (50 μl) of human SH-SY5Y neuroblastoma cell line stably expressing APPswe treated as described in *J*. DMSO-treated cells were used as control. Aβ was quantified using Aβ40 or Aβ42 standard curves. The graph represents the mean ± S.E. from three experiments and is expressed in pg/mg protein. *, *p* < 0.01 calculated *versus* DMSO treated SH-SY5Y APPswe cells using one-way ANOVA and Tukey's multiple comparisons test. *P*, representative SDS-PAGE analyses and quantification of RyR2 immunoprecipitated from the human SH-SY5Y neuroblastoma cell line stably expressing mock vector used as control (*Control*) or APPswe treated as described in *J*. RyR2 was immunoprecipitated and immunoblotted for RyR2-pS2808 (RyR2 PKA phosphorylation site), *S*-nitrosylation (*CysNO*), and oxidation (*DNP*) as well as calstabin2. *Q–T*, the graphs represent the mean ± S.E. obtained from three independent experiments. *, *p* < 0.05 calculated *versus* control; #, *p* < 0.05 calculated *versus* DMSO or vehicle using one-way ANOVA and Bonferroni post-test. *A.U.*, arbitrary units.

We then sought to determine the potential influence of Aβ or other APP metabolites on the RyR2 macromolecular complex remodeling. We used an Aβ preparation containing soluble Aβ oligomers (considered as the most toxic forms of Aβ) (Fig. 3*I* and Ref. 31) and found that exposure of control human SH-SY5Y neuroblastoma cells to Aβ (1–5 nm, 30–60 min) resulted in RyR2 PKA phosphorylation, oxidation, nitrosylation, and depletion of calstabin2 from the RyR2 macromolecular complex (Fig. 3, *A–E*). However, in contrast to SH-SY5Y-APPswe cells, the amounts of PP1 and spinophilin in the RyR2 complex were unaffected by acute treatment with Aβ (Fig. 3, *A*, *F*, and *G*). Pretreatment with S107 reduced Aβ-induced calstabin2 depletion from the RyR2 macromolecular complex (Fig. 3, *A* and *E*). We also show that treatment of SH-SY5Y control cells with increasing concentrations of the β-AR agonist isoproterenol induces RyR2 phosphorylation associated with a dissociation of calstabin2 from RyR2 (Fig. 2, *G* and *H*). ICI inhibited Aβ-induced RyR2 remodeling, thus preventing RyR2 PKA phosphorylation, oxidation, nitrosylation, and calstabin2 depletion (Fig. 3, *A–E*). Neither S107 nor ICI affected PP1 and spinophilin levels in the RyR2 complex. Acute Aβ exposure increased cAMP levels (14.2 ± 0.2 *versus* 8.0 ± 0.3 pmol/mg), which was inhibited by ICI (7.9 ± 0.1 pmol/mg) (Fig. 3*H*), suggesting that Aβ acts upstream of β2-AR signaling. These data and those of Fig. 1 demonstrate that RyR2 remodeling is likely linked to Aβ (Figs. 1 and 3, *A* and *H*) and to β2-AR activation (Fig. 2, *A–H*). Because our Aβ preparation contains monomeric and low molecular weight oligomeric forms of Aβ, we cannot attribute the observed effects to a specific Aβ species. We could detect only a moderate elevation of cAMP levels in APPswe model (∼20% increase over control cells considered as 100%, *n* = 3; data not shown), but data were obtained at only one time point. Thus, other experiments will be necessary to unravel time-dependent modulation of cAMP production in the APPswe model.

We also used a pharmacological approach to modulate APP metabolism by using β-secretase (LY288672, 30 μm) or γ-secretase (ELND006, 5 μm) inhibitors and investigate the potential implication of other βAPP metabolites in RyR2 remodeling.

Although inhibition of β-secretase reduces C99 fragment production and enhances the level of C83 fragment, γ-secretase inhibition enhances the levels of both C99 and C83 fragments (Fig. 3, *J* and *K*). As expected, β- and γ-secretase inhibitors fully block intracellular and extracellular Aβ production (Fig. 3, *L–O*). Interestingly, blocking β-secretase or γ-secretase reduces RyR2 PKA phosphorylation, oxidation, nitrosylation, and calstabin2 depletion in APPswe-expressing cells (Fig. 3, *P–T*).

### Aβ-mediated remodeling of RyR2 channel increases cytosolic Ca^2+^ and mitochondrial ROS in SH-SY5Y neuroblastoma cells

**Figure 4.**
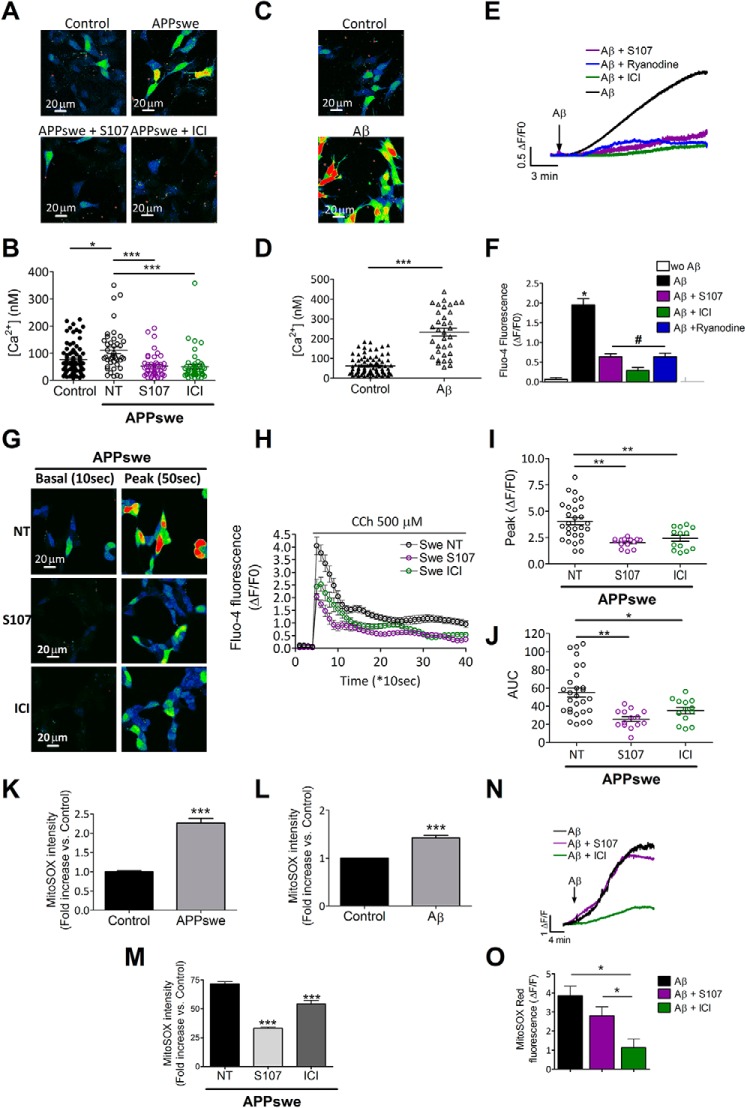
**Aβ-mediated treatment elevates basal cytosolic [Ca^2+^] and mitochondrial ROS in human neuroblastoma cells.** *A*, representative pseudo-colored images (projection of Z-stacks) of basal [Ca^2+^]_cyt_ in human SH-SY5Y neuroblastoma cell line stably expressing APPswe or mock vector used as control measured using Fluo-4, AM probe. The *scale bar* represents 20 μm. *B*, the scatter plot represents calibrated [Ca^2+^]_cyt_ (nm) obtained in control cells (*n* = 84) and in APPswe-expressing cells untreated (*n* = 42) or treated with S107 (1 μm, for 12 h) (*n* = 46) or with ICI (10 μm, for 12 h) (*n* = 47). *, *p* < 0.05; ***, *p* < 0.001, calculated using one-way ANOVA and Tukey's multiple comparisons test. *C*, representative pseudo-colored images (projection of Z-stacks) of basal [Ca^2+^]_cyt_ measured using Fluo-4, AM in the human SH-SY5Y neuroblastoma cell line treated with Aβ (1–5 nm) for 12 h. The *scale bar* represents 20 μm. *D*, the scatter plot represents calibrated [Ca^2+^]_cyt_ (nm) obtained in the control (*n* = 83) or Aβ-treated cells (*n* = 34). ***, *p* < 0.001 calculated *versus* control using the *t* test. *E*, acute Aβ application caused a rise in [Ca^2+^]_cyt_ in the SH-SY5Y neuroblastoma cell line (WT) (*n* = 42) that was partially inhibited by either S107 (10 μm) (*n* = 31), ICI (1 μm) (*n* = 44), or ryanodine (10 μm) (*n* = 23) pretreatment. *F*, the graph shows the mean ± S.E. of Δ*F*/*F*_0_ of Fluo-4 fluorescence in the plateau phase. *, *p* < 0.001 calculated *versus* control (*wo A*β); #, *p* < 0.001 calculated *versus* Aβ-treated cells using one way ANOVA and Tukey's multiple comparisons test. *G*, representative pseudo-colored images (focal plane) of the Fluo-4 signal before (10 s, basal) and after carbachol stimulation (500 μm) (50 s, peak) measured using Fluo-4 AM probe in SH-SY5Y neuroblastoma cell line stably expressing APPswe non-treated cells (*NT*) (*n* = 28) or treated for 12 h with either S107 (1 μm) (*n* = 14) or ICI (10 μm) (*n* = 13). The *color scale* is shown where the *black*/*blue* and the *yellow*/*red* represent low and high Fluo-4 fluorescence respectively. *H*, normalized curves showing carbachol (*CCh*)-mediated Ca^2+^ responses, presented as the mean Δ*F*/*F*_0_ ± S.E. *I*, scatter plots represent the mean of peak values presented as mean Δ*F*/*F*_0_ ± S.E. for non-treated (*NT*) SH-SY5Y APPswe (*n* = 28) or cells treated for 12 h with S107 (1 μm, *n* = 14) or ICI (10 μm, *n* = 13). *J*, scatter plots represent the mean area under the curve (*AUC*) ± S.E. units of the normalized Ca^2+^ response for cells as in *J. I* and *J*: *, *p* < 0,05; **, *p* < 0,01 analyzed by one-way ANOVA and Dunnett's post- test *versus* APPswe NT. *K–M*, the graphs represent MitoSOX red dye -fold change in control (*n* = 228) and APPswe cells (*n* = 144) (*K*) and in control (*n* = 6) and Aβ-treated (*n* = 6) cells (*N*), and in SH-SY5Y APPswe non-treated (*n* = 293) and treated with S107 (*n* = 382) or ICI (*n* = 136) (*M*). ***, *p* < 0.001 analyzed by Student's *t* test (*K* and *L*) and one-way ANOVA and Dunnett's post-test *versus* APPswe NT (*M*). *N* and *O*, acute Aβ treatment caused a rise in mitochondrial ROS production, as detected by MitoSOX red dye, in the SH-SY5Y neuroblastoma cell line (*n* = 293 for Aβ treated cells) that was not inhibited by S107 (*n* = 382) and reduced by ICI (*n* = 136). The graph shows the mean ± S.E. The graph shows Δ*F*/*F* of MitoSox fluorescence in the plateau phase normalized to the basal level before Aβ stimulation. *, *p* < 0.05 using one-way ANOVA and Dunnett's post-test *versus* Aβ.

We previously reported that SH-SY5Y cells expressing APPswe display increased cytosolic Ca^2+^ due to increased Ca^2+^ release from the ER via IP_3_R and RyR and enhanced Ca^2+^ entry via voltage-dependent and voltage-independent plasma membrane Ca^2+^ channels (13). We now report that SH-SY5Y cells expressing APPswe exhibit increased basal cytosolic Ca^2+^ signals compared with control cells (Fig. 4, *A* and *B*). Accordingly, exogenous Aβ treatment of SH-SY5Y cells for 12 h (an experimental condition mimicking APPswe cells chronically overproducing Aβ) also results in elevated basal [Ca^2+^]_cyt_ (Fig. 4, *C* and *D*). As previously reported (35), acute application of Aβ to human neuroblastoma SH-SY5Y cells induced a slow rise of [Ca^2+^]_cyt_ (Fig. 4, *E* and *F*). Interestingly, Aβ-induced Ca^2+^ elevation was prevented by S107, ICI, or ryanodine pretreatment (Δ*F*/*F*_0_ = 1.95 ± 0.16 (*n* = 57), 0.63 ± 0.08 (*n* = 31), 0.28 ± 0.08 (*n* = 44), 0.63 ± 0.09 (*n* = 23) in Aβ, Aβ+S107-, Aβ+ICI-, and Aβ+ryanodine-treated cells, respectively) (Fig. 4, *E* and *F*). Our finding is in accordance with data showing that preincubation with inhibitory ryanodine virtually eliminated the generation of Ca^2+^ signals elicited by oligomeric Aβ in primary hippocampal neurons (36).

S107, ICI, and ryanodine did not completely reverse the rise in [Ca^2+^]_cyt_. This may indicate that, in addition to leaky RyR2, there are other components that contribute to the Aβ-induced rise in [Ca^2+^]_cyt_. Possibilities include: 1) intracellular Ca^2+^ release via the IP_3_Rs and 2) Ca^2+^ entry through the plasma membrane as previously reported (13). Accordingly, Aβ-mediated Ca^2+^ elevation is lower in nominally Ca^2+^-free extracellular medium, and this elevation is completely reversed by ryanodine (data not shown).

We also analyzed basal [Ca^2+^]_cyt_ in APPswe cells untreated or treated with S107 or ICI (Fig. 4, *A* and *B*). We show that S107 or blocking β2-adrenergic signaling by ICI significantly reduce basal [Ca^2+^]_cyt_ in APPswe-expressing cells (Fig. 4, *A* and *B*).

It is known that IP_3_Rs are activated by Ins(1,4,5)P3, a metabolic product of GPCR activity, and that the activation of these channels is amplified by Ca^2+^-induced Ca^2+^ release, a regenerative mechanism by which Ca^2+^ enhances its own release from IP_3_Rs and RyRs (37). Accordingly, it was already reported that exacerbated IP_3_R-evoked Ca^2+^ signals in the PS1_M146V_- and the 3×Tg-AD-derived neurons occur through increased Ca^2+^-induced Ca^2+^ release through the RyR (15). We show herein that increased inositol 1,4,5-trisphosphate-mediated cytosolic Ca^2+^ signal (carbachol stimulation) in APPswe (Fig. 4, *G–J*) contributed to RyR-mediated Ca^2+^ release/leak. Thus, stabilization of calstabin2 on RyR2 channels by S107 or blockade of RyR2 phosphorylation by ICI (Fig. 4, *G0-J*), both, lowers carbachol-mediated cytosolic Ca^2+^ signals as revealed by reduced peak values and the area under the curve reflecting integrated Ca^2+^ response (Fig. 4, *G–J*). Similar results were obtained using cytosolic aequorin probe providing “calibrated” measurements of [Ca^2+^]_cyt_ (38) (supplemental Fig. S1). All over, these data demonstrate that cytosolic Ca^2+^ increase in APPswe cells and in Aβ-treated cells is largely contributed by RyR-mediated Ca^2+^ release.

Next we found that both endogenous production (*i.e.* APPswe cells) and exogenous application of Aβ (*i.e.* treatment of wild-type SH-SY5Y cells with Aβ for 12 h) resulted in increased mitochondrial reactive oxygen species (ROS) production (Fig. 4, *K* and *L*). Interestingly, mitochondrial ROS production stimulated by chronic Aβ production in SH-SY5Y-APPswe cells was partially inhibited by both ICI and S107 (Fig. 4*M*). We also show that acute application of Aβ enhanced MitoSOX fluorescence until reaching a steady state plateau (Fig. 4*N*). *Bar graphs* show the mean plateau value of Δ*F*/*F* ± S.E. obtained in each experimental condition and reveal a significant decrease of MitoSOX fluorescence intensity in Aβ+ICI cells *versus* Aβ alone and Aβ+S107 (Fig. 4*O*). S107 pretreatment reduced Aβ-mediated ROS production (plateau phase of representative data in Fig. 4*O*), but this reduction was not statistically significant. These data reveal that Aβ may contribute to mitochondrial ROS elevation that is significantly reversed by ICI and to a lesser extent with S107 (Fig. 4, *N* and *O*). These data may indicate that mitochondrial ROS elevation in APPswe likely results from both β-adrenergic and RyR-mediated Ca^2+^ leak. In Aβ-treated cells, mitochondrial ROS elevation likely occurs in a β-adrenergic-dependent manner and is only partially due to RyR-mediated ER Ca^2+^ leak.

### Pharmacological inhibition of RyR2 leak reduces βAPP metabolism and the production of Aβ in APPswe-expressing SH-SY5Y cells

**Figure 5.**
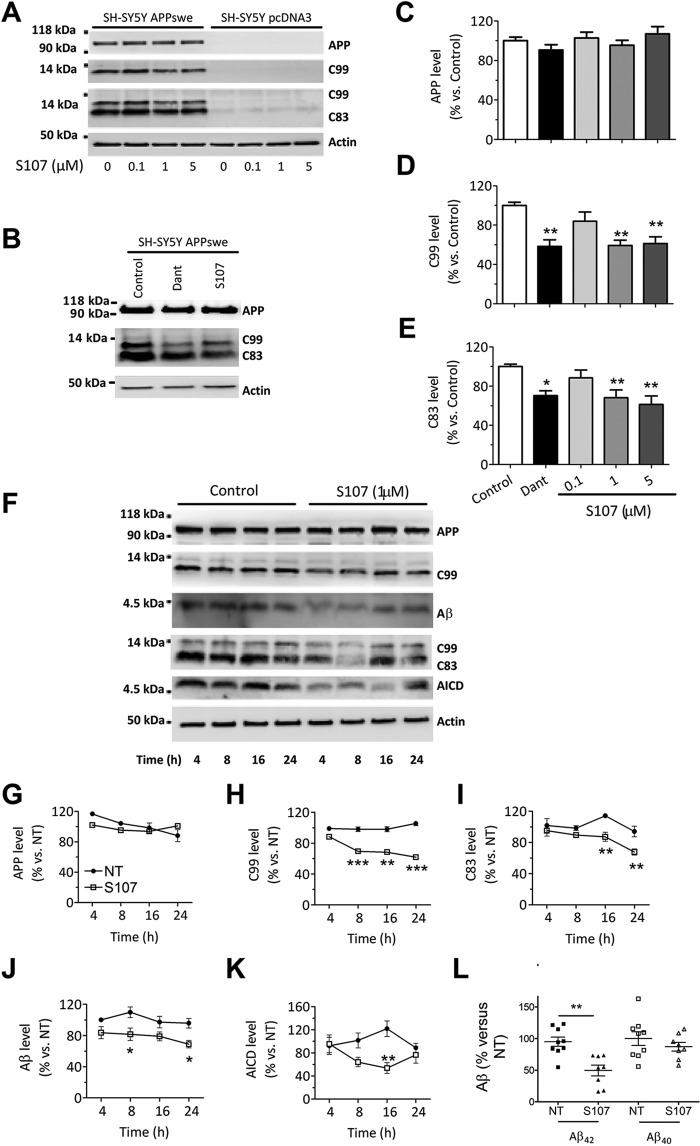
**Pharmacologic inhibition of RyR2 leak reduced βAPP metabolism and the production of CTFs and of Aβ in SH-SY5Y APPswe model.** *A*, representative Tris-Tricine and SDS-PAGE analyses of full-length βAPP, C83, and C99 levels (revealed as described in Fig. 2*J*) in SH-SY5Y cells expressing pcDNA3.1 or APPswe non-treated (0) or treated with S107 (0.1, 1, or 5 μm for 12 h). *B*, representative Tris-Tricine and SDS-PAGE analyses of full-length βAPP and its CTF (C99 and C83) in SH-SY5Y cells expressing APPswe non-treated (*Control*) or treated with dantrolene (50 μm) or S107 (1 μm) for 12 h. *C–E*, graphs show the mean ± S.E. obtained from control (*n* = 11), S107 (0.1, 1, or 5 μm, *n* = 5, 3, and 9, respectively), and dantrolene (*n* = 5). *, *p* < 0.05; **, *p* < 0.01, *versus* non-treated cells analyzed by one-way ANOVA and Dunnett's post-test *versus* APPswe control cells. *F*, representative Tris-Tricine and SDS-PAGE analyses of full-length βAPP, C83 and C99, Aβ, and AICD levels in SH-SY5Y cells expressing APPswe non-treated (*Control*) or treated with S107 (1 μm) for the indicated times (4, 8, 16, and 24 h). βAPP was detected using APP N-terminal antibody. C99 and Aβ were detected using 6E10 antibody. C83 and C99 and AICD were detected using APP C-terminal antibody. *A* and *E*, actin was used as loading control. *G-K*, graphs show the mean ± S.E. obtained from seven different experiments. *, *p* < 0.05; **, *p* < 0.01; ***, *p* < 0.001 *versus* controls at the same time of analysis using two-way ANOVA and the Bonferroni post-test. *L*, ELISA analyses of extracellular Aβ40 and Aβ42 in APPswe cells treated with S107 (1 μm, for 4 h) and presented as % *versus* APPswe control cells. **, *p* < 0.01 using *t* test, from 8 experiments. *NT*, non-treated cells.

**Figure 6.**
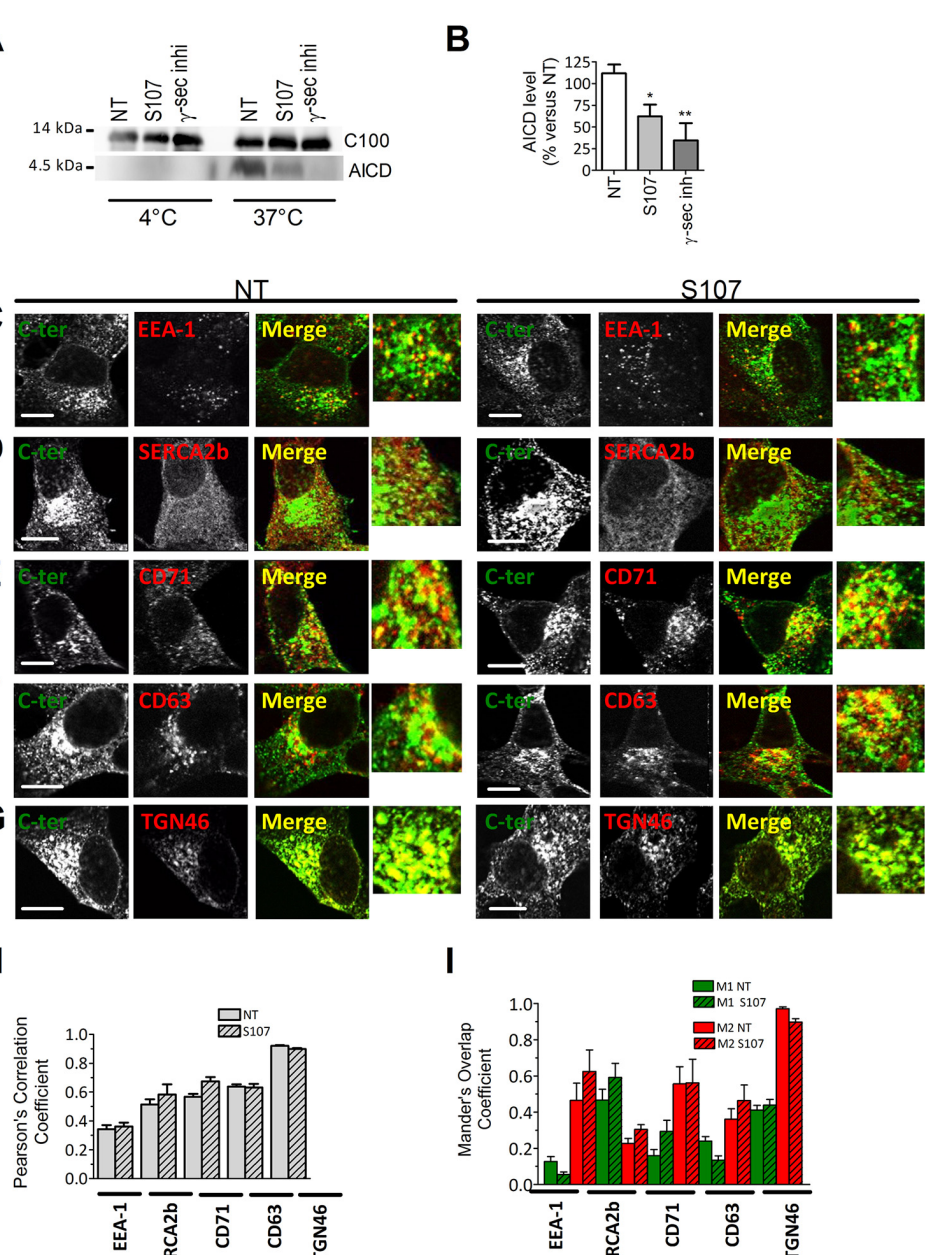
**Pharmacologic inhibition of RyR2 leak reduced γ-secretase activity and does not alter βAPP intracellular localization.** *A*, cell-free AICD production from recombinant C100 peptide performed at 37 °C or 4 °C in the presence of membrane fractions isolated from APPswe cells treated with S107 (1 μm, for 12 h) or with γ-secretase inhibitor (*inih*; 5 μm, for 12 h). C100 and AICD were detected using APP C-terminal antibody. *NT*, non-treated cells. *B*, graph shows the mean ± S.E. obtained from seven different experiments. *, *p* < 0.05; **, *p* < 0.01 *versus* APPswe non-treated cells using one-way ANOVA and Dunnett's post-test *versus* APPswe NT. *C–G*, double-labeling immunofluorescence of SH-SY5Y cells expressing APPswe non-treated or treated with S107 (1 μm) for 12 h using antibodies against C-terminal (C*-ter*) APP antibody (*green signal*) and EEA1 (*C*), Ca^2+^-ATPase of the endoplasmic reticulum (*SERCA2b*) (*D*), the recycling endosome marker transferrin receptor (*CD71*) (*E*), lysosome membrane and late endosome membrane (*CD63*) (*F*), and the trans-Golgi network marker (*TGN46*) (*G*). *Green* and *red* signals are depicted in *white* to have better contrast visualization. Merge and magnified overlay images show *green* and *red signals* co-localization (depicted as *yellow signals*). The *scale bar* represents 10 μm. *H* and *I*, the graphs show Pearson's correlation coefficient (*H*) and Mander's Overlap Coefficient (*I*). *M1* corresponds to the green signal overlapping with the red signal. *M2* corresponds to the red signal overlapping with the green signal. Analyses were performed in at least four different fields in each condition obtained from three independent experiments. *H* and *I*, differences are statistically non-significant.

Next, we determined whether S107-mediated inhibition of RyR2 leak could affect βAPP processing and Aβ production in SH-SY5Y cells expressing APPswe. Interestingly, the levels of C83 and C99 were reduced by S107 treatment (1–5 μm) (Fig. 5*A*, D, E, F, H, and *I*). As the control, we used dantrolene to block RyR-mediated Ca^2+^ release (Fig. 5, *B*, *D*, and *E*). C99 undergoes cleavage by γ-secretase to yield Aβ40 and Aβ42 peptides and AICD (39). AICD is also produced from C83 fragment upon γ-secretase cleavage (39). S107 treatment reduced the level of AICD (Fig. 5, *F* and *K*) and of total intracellular Aβ (Fig. 5, *F* and *J*). Interestingly, the total amount of secreted Aβ42, but not of Aβ40, was also significantly reduced by S107 treatment in SH-5Y5Y cells expressing APPswe (Fig. 5*L*). Thus, S107 treatment reduces γ-secretase-mediated AICD production in an APPswe-expressing cells membrane preparation, similar to γ-secretase inhibition (Fig. 6, *A* and *B*).

We also evaluated whether S107 affects βAPP trafficking (localization). This was performed using immunofluorescence and confocal imaging with antibodies recognizing specific cellular compartments involved in βAPP trafficking. The cellular localization of βAPP was assessed using Pearson's correlation and Mander's Overlap coefficients (Fig. 6, *H–I*). We show that S107 did not alter βAPP localization in early endosome (EEA1) (Fig. 6, *C*, *H*, and *I*), ER (SERCA2b) (Fig. 6, *D*, *H*, and *I*), recycling endosomes (CD71) (Fig. 6, *E*, *H*, and *I*), late endosomal and lysosomal membranes (CD63) (Fig. 6, *F*, *H*, and *I*), and the trans-Golgi network (TGN46) (Fig. 6, *G*, *H*, and *I*).

### Blockade of β2- but not β1-adrenergic signaling reduces βAPP metabolism in APPswe-expressing SH-SY5Y cells

**Figure 7.**
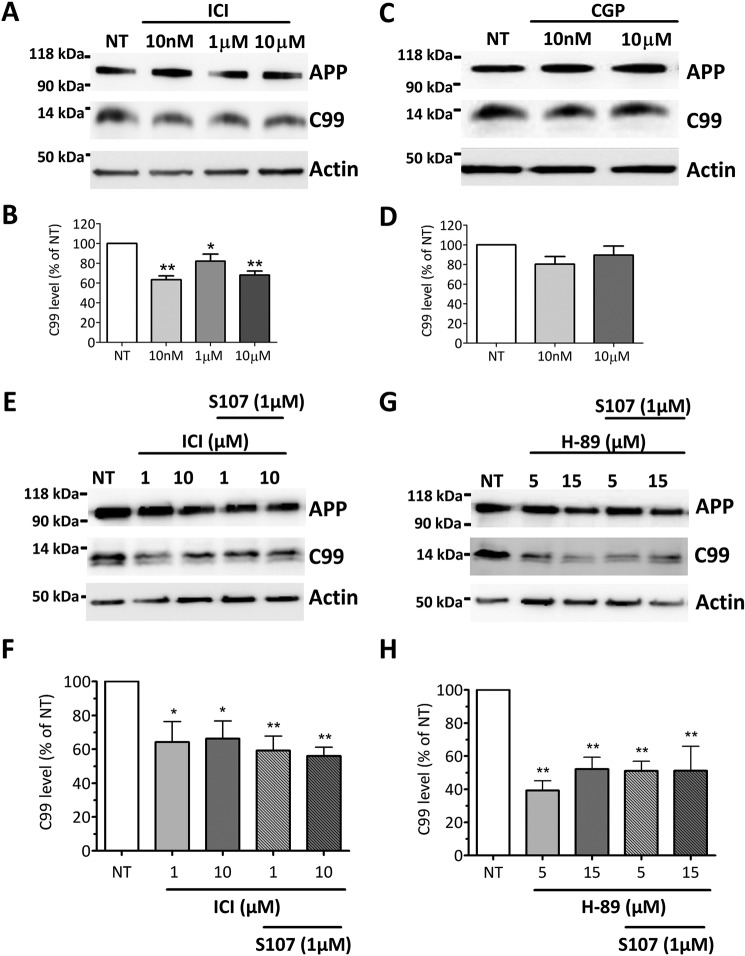
**Blockade of β2- but not of β1-adrenergic, cascade reduced βAPP metabolism in SH-SY5Y APPswe cells.** *A* and *C*, representative Tris-Tricine and SDS-PAGE analyses of full-length βAPP and C99 levels (determined as described in Fig. 5*A*) in SH-SY5Y cells expressing APPswe non-treated (*NT*) or treated with ICI (10 nm, 1 μm, or 10 μm) (*A*) or with CGP 20712A (10 nm or 10 μm) (*C*) for 12 h. *B* and *D*, graphs show the mean ± S.E. for ICI (*B*) and CGP (*D*) treatments, respectively, obtained from six different experiments. *, *p* < 0.05; **, *p* < 0.01 analyzed by one-way ANOVA and Dunnett's post-test *versus* APPswe NT. *E* and *G*, representative Tris-Tricine and SDS-PAGE analyses of full-length βAPP and C99 levels in SH-SY5Y cells expressing APPswe non-treated (*NT*), treated for 12 h with ICI (1 μm or 10 μm) (*E*) or H-89 (5 μm or 15 μm) (*G*) alone or in combination with S107 1 μm for 12 h. *F* and *H*, graphs show the mean ± S.E. obtained from five different experiments. *, *p* < 0.05; **, *p* < 0.01, analyzed using one-way ANOVA and Dunnett's post-test *versus* APPswe NT.

As with S107 treatment, blockade of β2-AR with ICI reduced the production of the C99 fragment in APPswe-expressing SH-SY5Y cells (Fig. 7, *A* and *B*), whereas the blockade of β1-AR with CGP did not (Fig. 7, *C* and *D*). The effect of ICI and S107 on C99 production was not additive (Fig. 7, *E* and *F*). In accordance with ICI data, inhibition of PKA by H-89 reduced [Ca^2+^]_cyt_ (supplemental Fig. S1), and C99 production (Fig. 7, *G* and *H*). Thus, RyR2 channel remodeling occurring downstream of β2-adrenergic signaling contributes to regulation of βAPP processing.

## Discussion

We show herein that RyR post-translational remodeling accounts for exacerbated RyR2-mediated Ca^2+^ release in an *in vitro* AD study model. Enhanced neuronal RyR2-mediated ER Ca^2+^ leak is linked to pathophysiological post-translational modifications in the macromolecular RyR complex (17). Interestingly, post-translational modifications of RyR2 were reported in cerebral ischemia (18), where endogenous RyRs undergo *S*-nitrosylation and *S*-gluthathionylation processes that resulted in high activity channels and ultimately lead to cortical neuronal death (18). Recently, Liu *et al.* (19) described the contribution of RyR2 post-translational remodeling to stress-related memory impairments.

We report that RyR2 undergoes PKA phosphorylation, oxidation, and nitrosylation in SH-SY5Y-overexpressing APPswe. Remodeled RyR2 macromolecular complex is depleted of calstabin2 and of both PP1 and its anchoring protein spinophilin. The equilibrium of phosphorylation and dephosphorylation of the channel is generally regarded as an important regulatory mechanism of RyR. Importantly, the amount of spinophilin (a postsynaptic marker) is decreased in the AD brain (40, 41). We report a depletion of PP1 and spinophilin in SH-SY5Y-overexpressing APPswe mutation producing chronically Aβ but not upon acute treatment of wild type SH-SY5Y with oligomeric Aβ. This may suggest that spinophilin depletion contributes to PKA phosphorylation of RyR2 by reducing the targeting of the phosphatase PP1 to the channel likely at advanced AD stages. We focused in this study on RyR2 and its associated regulatory protein calstabin2. We cannot exclude the possibility that RyR1 and RyR3 may also play a role in AD pathology.

**Figure 8.**
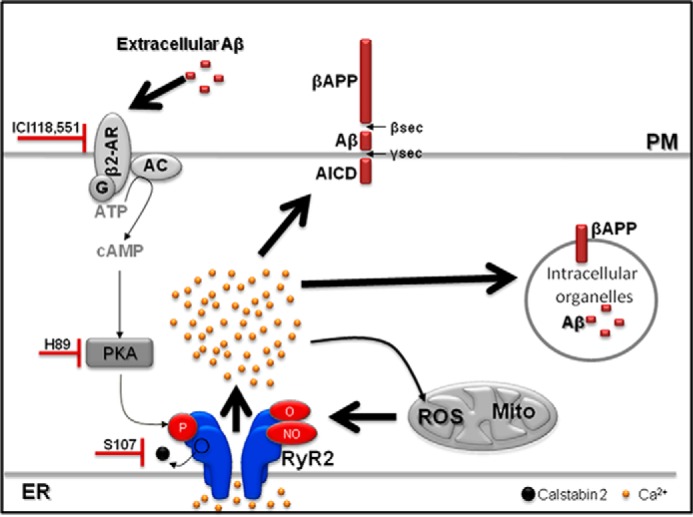
**Scheme showing the cascade linking Aβ, β2-adrenergic signaling, and leaky RyR2 channels in SH-SY5Y cells expressing APPswe.** βAPP processing occurs within intracellular organelles and in the plasma membrane (*PM*), thus producing Aβ inside intracellular organelles and in the extracellular media. Aβ-mediated β2-AR activation leads to RyR2 phosphorylation and calstabin2 dissociation, thereby enhancing RyR2-Ca^2+^ leak. RyR2-Ca^2+^ leak promotes Ca^2+^ entry into mitochondria (97) and mitochondrial ROS production. Mitochondrial ROS production leads to RyR2 oxidation and nitrosylation. Post-translational RyR2 remodeling (phosphorylation, oxidation, and nitrosylation) and calstabin2 dissociation enhances RyR2-Ca^2+^ leak. Blocking β2-adrenergic signaling or RyR2 channel leak reduces βAPP processing and Aβ production. *AC*, adenylate cyclase.

Post-translational modifications of RyRs in AD patients have not been reported yet. However, AD brains manifest excessive generation of reactive nitrogen and ROS, contributing to neuronal cell injury and death via a series of redox reactions (40, 42–44). cAMP levels are also significantly elevated in CSF from patients with Alzheimer dementia (45). In addition, PKA activation has been shown to contribute to the regulation of βAPP processing, to Aβ-mediated cell death *in vitro* and *in vivo*, and to oxidative stress (46–50). Interestingly, Aβ stimulates PKA activity and PKA-dependent signaling pathways by activating β-ARs (51–53). Blocking β2-adrenergic signaling diminishes Aβ production and delays functional decline in AD (47, 48, 54). Accordingly, activation of β2-ARs enhances γ-secretase activity and accelerates Aβ plaques formation (20, 22), a process that can be blocked by the specific β2-AR antagonist (22). Moreover, it was reported that Aβ42 peptide binds to the N terminus of β2-ARs, enhancing PKA-dependent AMPA receptor hyperactivity (55). Consistent with these observations, we show that acute application of Aβ to SH-SY5Y neuroblastoma cells increases cAMP levels and oxidative stress contributing to RyR2 remodeling that can be improved by treatment with β2-AR antagonist ICI. On the other hand, we show that both β2-AR antagonist ICI and PKA inhibitor H-89 reduce ER Ca^2+^ release and βAPP processing. This supports a positive feedback loop where Aβ activates β2-AR signaling cascade leading to RyR2 remodeling and ER Ca^2+^ leak that, in turn, amplifies βAPP processing and Aβ production (Fig. 8). We show that the increase of Aβ levels is linked to β2-AR signaling cascade. However, we cannot exclude the possibility that other G_s_-coupled receptors in PKA/cAMP pathway may also play a role in AD. It has previously been demonstrated that isoproterenol, a β-adrenergic agonist, increases mitochondrial ROS production in cardiomyocytes in a concentration- and cAMP-PKA-dependent manner (56). Mitochondrial ROS production could contribute to RyR2 oxidation. RyR2 leak may in turn increase mitochondrial ROS production (57). This seems to be the scenario in SH-SY5Y-expressing APPswe cells, as both Ca^2+^ and ROS elevation was reversed by S107 and ICI. The situation in acute Aβ-treated cells seems different as mitochondrial ROS elevation was reversed by the β2-AR antagonist and was only slightly contributed by leaky RyR channels (positive feedback loop in Fig. 8).

A growing body of literature supports the clinical relevance of the locus coeruleus (LC), norepinephrine, and noradrenergic receptors in AD as the LC widespread projections terminate in areas important for learning and memory such as the hippocampus and the cortex. Neuronal cell death in the LC and other brain stem nuclei is a well defined characteristic of AD pathology (58). It has been proposed that the downstream consequences of LC degeneration are decreased levels of norepinephrine in terminal regions (59) and a compensatory up-regulation of adrenergic receptors (60). A synthesis of evidence suggests that LC destruction may contribute to reduced Aβ clearance (58, 61, 62). These studies support our data showing that activation of the β-AR cascade occurs in a cell model of AD and that this contributes to enhanced Aβ production at least in part through PKA-mediated RyR remodeling and Ca^2+^ leak (Fig. 8).

Targeting of β2-adrenergic signaling to reverse/prevent AD pathology remains a controversial therapeutic track. Genetic knockdown or pharmacologic blockade of β2-AR elicits beneficial effects on taupathies (63) as well as in Tg2576 transgenic mice (64). Conversely, other studies have reported that administration of a β2-AR antagonist exacerbates neuropathology and cognitive deficits in a mouse model of AD (65) and that pharmacological stimulation of β2-AR improves cognitive function and restores synaptic density in a mouse model of Down syndrome (66). Recently it has also been reported that an enriched environment activates β2-ARs and protects against Aβ-induced reduction in LTP and prevents hippocampal dysfunction by Aβ oligomers (67). PKA activation under adrenergic stimulation is important for learning and memory consolidation. It appears, however, that Aβ-mediated activation of this cascade may be detrimental.

Increased [Ca^2+^]_cyt_ was observed by different laboratories in various AD models (68–72). Importantly, elevated basal Ca^2+^ has also been reported in peripheral blood mononuclear cells of AD patients (73). Our results contradict a previous study showing that transient expression of FAD APP mutations does not directly perturb intracellular Ca^2+^ homeostasis (74). The level and nature of βAPP metabolites (*i.e.* in our model stably expressing βAPP and the former study transiently expressing FAD APP mutants; Ref. 74) could explain such discrepancies. Altered RyR-mediated ER Ca^2+^ increase has also been observed in non-APP models such as PS1 and PS2 mutant mice and cell models (15, 75–78). However, post-translational remodeling of RyRs has not been reported in PS1/2 models. Nevertheless, co-immunoprecipitation has revealed a physical interaction between PS1–2 and RyR2 (79–81). Other recent studies demonstrated that the interaction of RyR with PS1 and PS2 N-terminal fragments strongly increased both mean currents and open probability of single RyR channels in a Ca^2+^-dependent manner (82, 83). Other groups demonstrated the molecular interaction of PS2 and sorcin (a modulator of RyR channel) (84).

These studies provide evidence that PS may also play an important role in the regulation of RyR channel activity. However, the question remains whether this regulation occurs only under physiological conditions or may also occur under pathological conditions, namely in cells expressing the mutated forms of PS1–2. Also unclear is whether β-AR-mediated RyR post-translational remodeling occurs in non-APP models, such as PS1 and PS2 mutant mice and cell models.

It has been reported that Ca^2+^ plays a role in the production of Aβ peptides (for review, see Ref. 9). Our data show that inhibiting RyR-mediated Ca^2+^ leak either with S107 or upstream by blocking β2-adrenergic signaling pathway reduces βAPP metabolism and Aβ production. In support of these findings the RyR active drug dantrolene has been shown to reduce the extent of βAPP phosphorylation likely through CdK5 and/or GSK3β Ca^2+^-dependent activation (13, 85). RyR-mediated Ca^2+^ leaks may also enhance β- and γ-secretase activities through direct interaction with these enzymes (86, 87) or by a stabilized γ-secretase complex (86, 87). Accordingly, we show that pharmacological blockade of Ca^2+^ leak by S107 reduces γ-secretase activity. These data led us to conclude that RyR post-translational remodeling amplifies AD pathogenesis through increased γ-secretase-mediated βAPP processing. The findings in the present study demonstrate that Aβ activates both β-adrenergic and oxidative stress. This leads to post-translational remodeling of RyR2 by PKA phosphorylation, oxidation, and nitrosylation and depletion of regulatory proteins calstabin2, PP1, and spinophilin. Data from this study and others (13, 36, 88, 89) are in agreement with a vicious cycle in which leaky RyR2 channels promote Aβ production and Aβ enhances RyR2 leak (Fig. 8). These data demonstrate that RyR channels could be envisioned as relevant candidates for a novel therapeutic approach for AD.

## Experimental procedures

### SH-SY5Y APPswe model and treatments

Human SH-SY5Y neuroblastoma cells (CRL-2266, ATCC) were cultured following the manufacturer's instructions. SH-SY5Y cells stably expressing empty pcDNA3.1 vector (Control) or pcDNA3.1 bearing APPswe cDNA were generated as already described (13) and maintained in the presence of Geneticin (400 μg, Gibco). In this study we used polyclonal stable cells.

Cells were treated overnight for 12 h with β- or γ-secretase inhibitors. γ-Secretase inhibitor ELND006 was used at a 5 μm final concentration and vehicle (methylcellulose/polysorbate 80, Sigma) was used as control (33, 34). β-Secretase inhibitor (Eli Lilly inhibitor LY288672 (32), synthesized, and kindly provided by Elan Pharmaceuticals) was used at 30 μm final concentration prepared in DMSO. In some experiments vehicle or DMSO were used as controls because no difference was observed between these two treatments. Cells were treated with various concentrations of: S107 (a benzothiazepine derivative) (19, 26, 28) (0.1, 1, 5, or 10 μm) for the indicated times (4, 8, 12, 16, or 24 h); H-89 2HCl (Sigma) (1 nm, 1 μm, 5 μm, or 15 μm), a PKA inhibitor; ICI 118,551, hydrochloride ((±)-1-[2,3-(dihydro-7-methyl-1H-inden-4-yl)oxy]-3-[(1-methylethyl) amino]-2-butanol hydrochloride) (Sigma) (1 nm, 10 nm, 100 nm, 1 μm, or 10 μm), a highly selective β2-adrenoreceptor antagonist; CGP, 20712A, ((±)-2-hydroxy-5-[2-[[2-hydroxy-3-[4-[1-methyl-4-(trifluoromethyl)-1H-imidazol-2-yl]phenoxy]propyl] amino]ethoxy]-benzamide methanesulfonate salt), a highly selective and potent β1-adrenoreceptor antagonist (Sigma) (1 nm, 10 nm, 100 nm, 1 μm, or 10 μm); isoproterenol hydrochloride ((−)-isoprenaline hydrochloride, (−)-*N*-isopropyl-l-noradrenaline hydrochloride, (*R*)-3,4-dihydroxy-α-(isopropylaminomethyl)benzyl alcohol hydrochloride), a β-adrenergic agonist (Sigma) (1 nm, 10 nm, 100 nm, or 1 μm). ICI, CGP, and H-89 were applied for 12 h. Isoproterenol was applied for 1 h.

Mock-transfected or APP695_LDN_-expressing CHO cells (individualized clones) were obtained by stable transfection of pcDNA4 empty vector (Control) and hAPP695 cDNA harboring London mutation (APP_LDN_: APPV642I) and subcloned in pcDNA4 vector. Cells were maintained in DMEM containing 10% FBS, sodium hypoxanthine-thymidine supplement, and 300 μm proline (31).

### Aβ preparation

Mock-transfected or APP695_LDN_ CHO cells were grown in 150-mm-diameter dishes until reaching 80% confluence, then washed with PBS and allowed to secrete for 24 h into 15 ml of Neurobasal medium (Invitrogen). Secretions were centrifuged (1000 × *g* for 10 min) and then concentrated into Amicon Ultra-15 3000 filters (4000 × *g* for 30 min). One-milliliter aliquots of Aβ preparation concentrates were stored at −80 °C until use. Aβ preparations were controlled on Tris-Tricine gels as already described (see the representative blot in Fig. 2*I*, and Ref. 31). This Aβ preparation yields both monomeric and oligomeric Aβ species but has the advantage of being a “natural” source of Aβ produced through sequential cleavage of βAPP.

### Aβ40 and Aβ42 peptide measurements

Detection of Aβ40 and Aβ42 peptides secreted in cell media were measured by an ELISA kit (NOVEX^TM^, ThermoFisher Scientific, France) using human C-terminal Aβ antibodies.

### Biochemical analyses of RyR2 channel remodeling

RyR2 was immunoprecipitated from cell lysates with an RyR2-specific antibody (2 μg) in 0.5 ml of a modified radioimmune precipitation assay buffer (50 mm Tris-HCl, pH 7.2, 0.9% NaCl, 5.0 mm NaF, 1.0 mm Na_3_VO_4_, 1% Triton X-100, and protease inhibitors) overnight at 4 °C. The RyR2-specific antibody is an affinity-purified polyclonal rabbit antibody custom-made by Yenzym Antibodies (San Francisco, CA) using the peptide CKPEFNNHKDYAQEK corresponding to amino acids 1367–1380 of mouse RyR2 with a cysteine residue added to the N terminus. The immune complexes were incubated with protein A-Sepharose beads (Sigma) at 4 °C for 1 h, and the beads were washed three times with radioimmune precipitation assay buffer. The immunoprecipitates were size-fractionated on SDS-PAGE gels (6% for RyR, 15% for calstabin) and transferred onto nitrocellulose membranes for 2 h at 200 mA. Immunoblots were developed using the following primary antibodies: anti-RyR2 (Affinity Bioreagents, 1:2000), anti-phospho-RyR-Ser(P)-2808 (1:5000; Ref 90), anti-calstabin (FKBP12 C-19, 1:1000, Santa Cruz Biotechnology, Inc., Santa Cruz, CA), anti-Cys-NO (1:1,000, Sigma), anti-PP1 (Santa Cruz Biotechnology, 1:2500), and anti-Spinophilin (Abcam, 1:500). To determine channel oxidation, the carbonyl groups in the protein side chains were derivatized to DNP by reaction with 2,4-dinitrophenylhydrazine. The DNP signal associated with RyR was determined using a specific anti-DNP antibody according to the manufacturer's instructions (Millipore, Billerica, MA). We did not explore other RyR2 phosphorylation sites responsive to other kinases (*i.e.* Ca^2+^/calmodulin-dependent kinase (CaMKII)). All immunoblots were developed with the Odyssey system (LI-COR Biosciences, Lincoln, NE) using IR-labeled anti-mouse and anti-rabbit IgG (1:10,000 dilution) secondary antibodies.

### Immunofluorescence analysis

Cells grown on 25-mm round coverslips were fixed in paraformaldehyde 4% solution for 10 min at room temperature. Cells were permeabilized with Triton 0.5%, and nonspecific binding sites were blocked for 1 h with BSA (3%). Cells were then incubated at 4 °C overnight with primary antibodies diluted in BSA (3%). After 3 washes, coverslips were incubated with secondary antibodies (fluorescent Alexa Fluor antibodies, Alexa 488- and Alexa 594-conjugated (Invitrogen; 1:1000)) at room temperature for 1 h. Immunofluorescence images were acquired on a Leica SP5 confocal microscope using excitation filters 488 and 594 nm. Images were analyzed using ImageJ software. Images were background-corrected, and the colocalization of red and green staining was determined using JACoP plugging. Pearson's correlation coefficient and Mander's Overlap Coefficient were used to evaluate the extent of colocalization as described (91).

We used βAPP C-terminal antibody (Sigma) recognizing 676–695 residues of βAPP. Other antibodies recognizing the following proteins were: SERCA2b antibody (clone IID8, Thermo Scientific Pierce Products) targeting SR Ca^2+^-ATPase of the endoplasmic reticulum; EEA1 antibody (clone 14/EEA1, BD Biosciences) targeting early endosome antigen 1; CD63 antibody (clone CLB-180, Abcam) targeting antigens of lysosome membrane and late endosome membrane; CD71 antibody (clone H68.4, Thermo Fisher Scientific) targeting transferrin receptor: TGN46 antibody (AHP500G, AbD Serotec).

### Measurements of mitochondrial superoxide concentration

We used MitoSOX (Invitrogen) Red fluorogenic dye to detect superoxide in the mitochondria of living cells (92). Cells grown on 24-mm coverslips were loaded with 5 μm MitoSOX red prepared in a KRB (containing 125 mm NaCl, 5 mm KCl, 1 mm Na_3_PO_4_, 1 mm MgSO_4_, 5.5 mm glucose, and 20 mm HEPES, pH 7.4) supplemented with 1 mm CaCl_2_ (KRB/CaCl_2_) at 37 °C for 30 min. After a brief wash, Z-stack images were acquired on a SP5 confocal microscope (Leica). Dye intensity was quantified on Z-stack projection of images after thresholding using Leica SP5 confocal microscope and ImageJ software. Atpenin A5, a specific potent inhibitor of mitochondrial respiratory chain complex II, was used as the positive control (data not shown) (93). We presented data as fluorescence intensity (-fold increase *versus* respective controls in each experiment). Kinetic measurements of MitoSOX are presented as Δ*F*/*F* to measure Aβ-mediated MitoSOX elevations correcting for differences in basal fluorescence or probe loading.

### Cytosolic Ca^2+^ measurements

Cells grown on 24-mm coverslips were loaded with 5 μm Fluo-4, AM (cytosolic Ca^2+^ probe), prepared in a KRB/CaCl_2_ at 37 °C for 30 min. After a brief washout, Z-stack images were acquired on a SP5 confocal microscope (Leica). Cellular loading of dyes was quantified on maximal projection of Z-stack images after thresholding using ImageJ software.

We calibrated the fluorescence measurements, and the intensities of fluorescence for each condition were translated into nanomolar concentrations of Ca^2+^ according to the formula,

(Eq. 1) $$\left[C{\text{a}}^{2+}\right]=Kd\left(\frac{F-F_{\min}}{F_{\max}-F}\right)$$

where *K_d_* for Fluo4 is 345 nm, *F* is the background-corrected fluorescence intensity recorded from cells during the experiment, *F*_min_ is the Ca^2+^-free indicator fluorescence (determined in the ionophore (ionomycin 0.5 μm)/40 mm MnCl2 solution), and *F*_max_ is the Ca^2+^-saturated indicator fluorescence (determined in the ionophore (ionomycin 0.5 μm)/1 mm Ca^2+^ rich solution) (94).

Kinetic measurement of Fluo4, AM fluorescence (Fig. 3, *E*, *F*, *H*, *I*, and *J*) changes were normalized and presented as Δ*F*/*F*_0_ to measure Aβ- or carbachol-mediated Ca^2+^ elevations correcting for differences in basal fluorescence or probe loading (95).

### cAMP measurement

We used the cAMP direct immunoassay kit (Abcam) following the manufacturer's instruction.

### βAPP processing analysis

To detect CTFs (C99 and C83) and Aβ peptide, protein extracts (40 μg) were incubated with 70% formic acid (Sigma) and SpeedVac-evaporated for 40 min. The pellets were dissolved in 1 m Tris, pH 10.8, 25 mm betaine and diluted in 2× Tris-Tricine loading buffer (125 mm Tris-HCl, pH 8.45, 2% SDS, 20% glycerol, 0.001% bromphenol blue, and 5% β-mercaptoethanol). Proteins were resolved by 16.5% Tris-Tricine SDS-PAGE and transferred onto PVDF membranes. Membranes were boiled in PBS and incubated overnight with specific antibodies.

### Antibodies

Aβ, β-CTF (C99), and full-length βAPP were detected using 6E10 antibody (Covance, Rueil-Malmaison, France), which recognizes 1–16 residues of Aβ. α- and β-APP CTFs (C83 and C99, respectively) were detected using the APP C-terminal antibody (Sigma) recognizing 676–695 residues of βAPP. Full-length βAPP was also detected using APP (N-terminal) antibody (22C11, Millipore, S.A.S. France). β-Actin (Sigma) was used as loading control.

### In vitro γ-secretase assay

*In vitro* γ-secretase activity was assessed as described (13, 96). 20 μg of each subcellular fraction were resuspended in solubilization buffer (150 mm sodium citrate, pH 6.4, containing 3-[(3-cholamydopropyl) and dimethylammonio]-2-hydroxy-1-propanesulfonate 1% (v/v) supplemented with protease inhibitor mixture. All steps were performed at 4 °C. Solubilized membranes were diluted once with sodium citrate buffer (150 mm pH 6.4) and with reaction buffer (150 mm sodium citrate, pH 6.4, 20 mm dithiothreitol, 0.2 mg/ml BSA, 1 mg/ml egg phosphatidylcholine and 50 μg/ml recombinant C100-FLAG). The resulting reaction mixtures were then either incubated with constant agitation for 16 h at 37 °C or stored at 4 °C (negative controls). Samples were then supplemented with 2× Tris-Tricine loading buffer, boiled for 5 min, and subjected to 16.5% Tris-Tricine SDS-PAGE.

### Statistical analyses

Data are expressed as the mean ± S.E. Sample size for each experiment is stated in the figure captions. Statistical analyses were performed using one-way or two-way ANOVA and Dunnett's, Bonferroni's, or Tukey's post-tests or *t* test. Minimum statistically significant differences were established at *p* < 0.05. Non-statistically significant differences are not shown in the graphs.

## Author contributions

A. R. M., A. L., and M. C. independently conceived the study. R. B., F. C., M. C., A. R. M., A. L., X. L., and S. R. designed the experiments. S. R. and V. S. conducted all the biochemistry assays of RyR2. R. B., M. C., and C. M. conducted the biochemistry assays of APP processing, immunofluorescence, and γ-secretase activity experiments. R. B., M. C., A. L., and X. L. conducted the Ca^2+^ experiments. M. C., A. L., X. L., and R. Z. analyzed ROS production measurements. A. R. M., M. C., F. C., and A. L. analyzed the data and wrote and edited the manuscript.

## Supplementary Material

Supplemental Data

## Footnotes

5 The abbreviations used are:

AD: Alzheimer disease
Aβ: amyloid β
βAPP: β-amyloid precursor protein
AICD: APP intracellular domain
β2-AR: β2-adrenergic receptor
CTF: C-terminal fragments
PP1: serine/threonine protein phosphatase 1
RyR: ryanodine receptor
SERCA: sarco-endoplasmic reticulum Ca^2+^-ATPase
C99: C-terminal fragment of 99 amino acids
C83: C-terminal fragment of 83 amino acids
PS: presenilin
ER: endoplasmic reticulum
IP_3_R: inositol 1,4,5-trisphosphate
ICI: ICI118–551
ROS: reactive oxygen species
EEA1: early endosome antigen 1
LC: locus coeruleus
CGP 20712A: (±)-2-hydroxy-5-[2-[[2-hydroxy-3-[4-[1-methyl-4-(trifluoromethyl)-1H-imidazol-2-yl]phenoxy]propyl] amino]ethoxy]-benzamide methanesulfonate salt
ICI 118,551: hydrochloride ((±)-1-[2,3-(dihydro-7-methyl-1H-inden-4-yl)oxy]-3-[(1-methylethyl)amino]-2-butanol hydrochloride
Tricine: *N*-[2-hydroxy-1,1-bis(hydroxymethyl)ethyl]glycine
ANOVA: analysis of variance
DNP: 2,4-dinitrophenylhydrazone.
