# Supplementary material for: Amyloid β production is regulated by β2-adrenergic signaling-mediated post-translational modifications of the ryanodine receptor
Source: J Biol Chem. 2017 May 5;292(24):10153–68. doi: 10.1074/jbc.M116.743070 (PMC5473221; doi:10.1074/jbc.M116.743070)
Supplement: Supplemental Data [file supp_292_24_10153__index.html]

Amyloid β production is regulated by β2-adrenergic signaling-mediated post-translational modifications of the ryanodine receptor — Amyloid β production is regulated by β2-adrenergic signaling-mediated post-translational modifications of the ryanodine receptor — RyR post-translational modifications enhance Aβ production — Supplemental Data 

# Amyloid β production is regulated by β2-adrenergic signaling-mediated post-translational modifications of the ryanodine receptor

## Supplemental Data

- Supplememental Fig S1 (.docx, 117 KB) - Supplemental Fig S1
