# Supplementary material for: Amyloid β production is regulated by β2-adrenergic signaling-mediated post-translational modifications of the ryanodine receptor
Source: J Biol Chem. 2017 May 5;292(24):10153–68. doi: 10.1074/jbc.M116.743070 (PMC5473221; doi:10.1074/jbc.M116.743070)
Supplement: Supplemental Data [file 10.1074_M116.743070_jbc.M116.743070-1.docx]

**
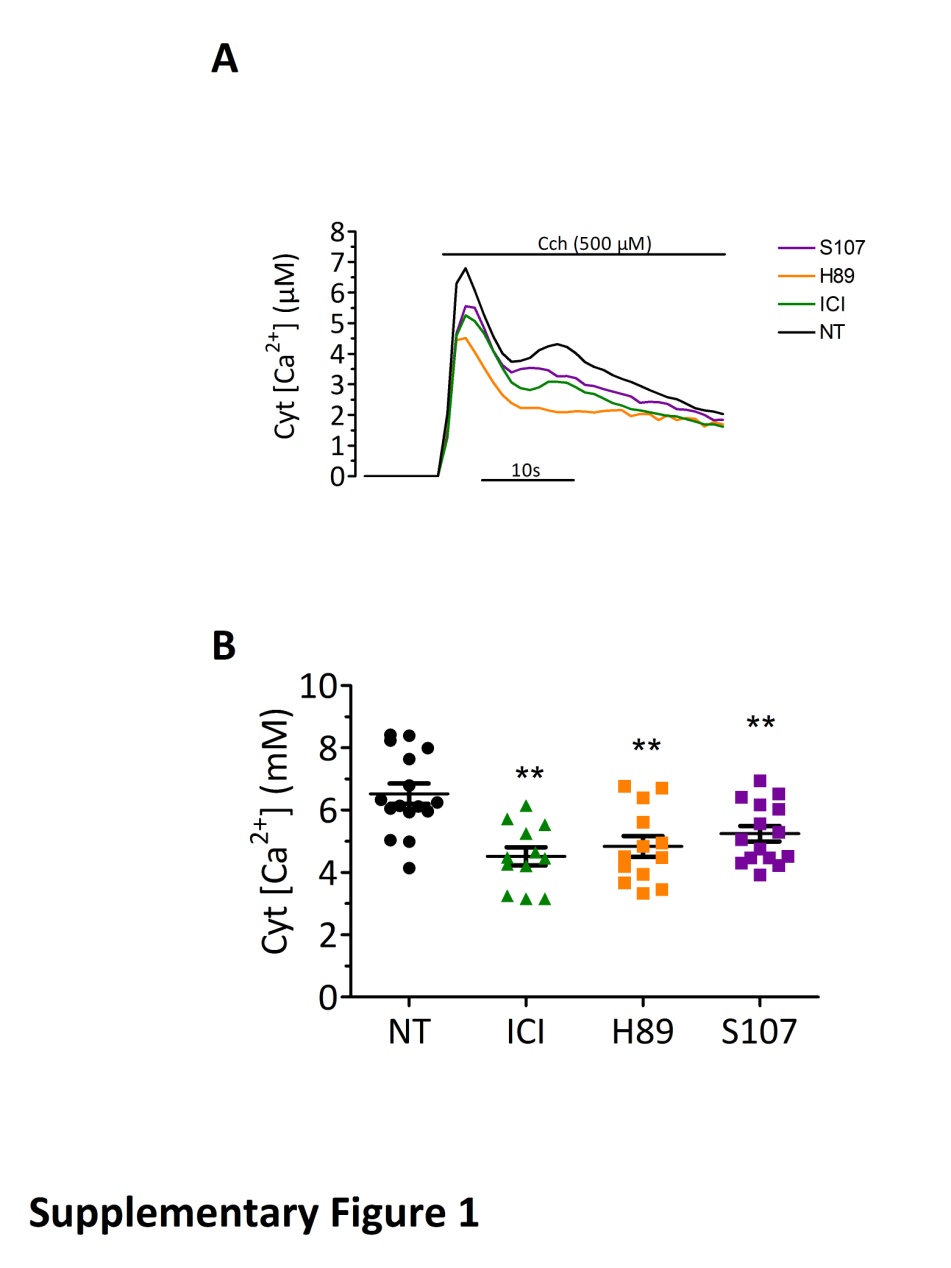
**

**Supplemental Figure S1**

To measure agonist-evoked cytosolic Ca^2+^ signals, we used the previously described adenoviral system expressing cytosolic-(AdCMVcytAEQ). Approximately 60000 cells were spotted on 13-mm coverslips, and infected 24hrs later with AdCMVcytAEQ (10^5^ infectious particle/ml). Aequorin measurements were performed 48hrs post-infection as already described (92). Cytosolic signals were obtained upon application of 500 µM Carbachol. Aequorin measurements were carried out in a plate reader (Varioskan, Thermoscientific). (A) representative curves showing carbachol-mediated Ca^2+^ responses in human SH-SY5Y neuroblastoma cell line stably expressing APPswe non treated (NT) or treated for 12hrs with S107 (1µM), ICI118-551 (ICI) (1µM), or H-89 (5µM). (B) Scatter plot represents Ca^2+^ peak values obtained upon Cch stimulation in control (n=16), S107 (n=15), and ICI (n=12) treated cells. ***p*< 0.01 calculated versus control using one-way ANOVA and Dunnett’s post-test calculated versus NT.
